# Molecular Mechanism of Biofilm Locator Protein Kinase Dbf2p-related kinase 1 in Regulating Innate Immune Response to Interleukin 17-induced Viral Pneumonia

**DOI:** 10.1080/21655979.2021.1996316

**Published:** 2021-12-06

**Authors:** Haifeng Wang, Lina Bu, Fang Shu, Yun Bai, Feixiao Xue, Shanshan Shi, Daqing Sun

**Affiliations:** a Department of Laboratory, Xi’an No.3 Hospital, The Affiliated Hospital of Northwest University, Xi’an, Shaanxi, P.R.China; b Department of Respiratory Medicine, Xi’an No.3 Hospital, The Affiliated Hospital of Northwest University, Xi’an, Shaanxi, P.R.China; c Department of Pediatric, The Affiliated Hospital of Northwest University, Xi’an, Shaanxi, P.R.China

**Keywords:** NDR1, IL17, respiratory syncytial virus, RAW264.7, inflammatory responses, immune response

## Abstract

It focused on the antiviral immune regulation of biofilm-localized protein kinase Dbf2p-related kinase 1 (NDR1) in viral pneumonia. Mouse alveolar monocyte RAW264.7 was used as blank control, and viral pneumonia cell model was prepared by infecting cells with respiratory syncytial virus (RSV). NDR1 overexpression vector and siRNA interference sequences were synthesized, and overexpression/silence NDR1 cell model was fabricated. About 50 ng/mL interleukin 17 (IL-17) was given to stimulate. Enzyme-linked immunosorbent assay (ELISA), quantitative reverse transcription PCR (RT-qRCR), and Western blot were performed to detect cytokines and chemokines, mRNA of inflammatory factors, and signal molecule protein expression. Notably, RSV infection increased RSV-F mRNA in RAW264.7 cells and reduced NDR1 mRNA and protein. Secretion levels of IL-6, interferon β (IFN-β), chemokine (C-X-C motif) ligand 2 (CXCL2), and chemokine (C-C motif) ligand 2 (CCL20) increased in the model group versus blank control (*P*< 0.05). IL-6, IFN-β, tumor necrosis factor α (TNF-α), and toll-like receptor 3 (TLR3) mRNA were up-regulated (*P < *0.05). Extracellular signal-regulated kinase (ERK1/2), p38 protein phosphorylation, human recombinant 1 (TBK1), and nuclear factor kappa-B (NF-κB) protein levels increased (*P < *0.05). After overexpression of NDR1, the secretion levels of cytokines and chemokines, inflammatory factors mRNA, and signal molecule protein increased significantly. After NDR1 was silenced, cytokines and chemokines, inflammatory factors mRNA, and signal molecule protein were not significantly different versus blank control group (*P > *0.05). In short, NDR1 regulated innate immune response to viral pneumonia induced by IL-17, which can be used as a new target for the treatment of IL-17-induced inflammatory response and autoimmune diseases.

## 1. Introduction

Influenza viral pneumonia refers to a serious interstitial pneumonia arising from viral infection and has the characteristics of high morbidity, rapid progress, and high mortality [1]. Respiratory syncytial virus (RSV) is a single-stranded negative-stranded RNA virus with a non-fragmented envelope. It belongs to the genus of pneumovirus in the paramyxovirus family. It can cause lower respiratory tract infections in children and the elderly [2]. RSV is often used in the preparation of viral pneumonia cell models [3]. Inflammation response is an important immune defense mechanism in the body, which can help the body resist infection by pathogenic microorganisms. Studies have confirmed that, when the virus invades the body and is not cleaned up in time, an excessive inflammatory immune response will occur in patients with influenza virus pneumonia, which will then cause substantial lung damage [4,5].

Virus infection is a dangerous factor that seriously threatens human life and health, and mutations and unknown virus infections have brought huge challenges to the fields of contemporary medicine and life sciences. Interleukin 17 (IL-17) is a pro-inflammatory cytokine secreted by Th17 cells [6]. Studies have shown that IL-17 plays an important role in a variety of autoimmune diseases, such as rheumatoid arthritis [7]. When foreign pathogens invade the body, IL-17 can induce the expression of inflammatory factors [8]. Dbf2p-related kinase 1 (NDR1) in the biomembrane-localized protein kinase belongs to the family of NDR-LATS protein kinases. It can positively regulate centrosome replication in a non-dependent manner, and participates in cell apoptosis, cycle regulation, and biological processes, such as autophagy [9]. Studies have confirmed that, when pathogenic microorganisms invade, inducing the expression of NDR1 can enhance the body’s resistance to pathogenic microorganism infection [10].

Above, NDR1 can regulate the body’s immune response caused by virus infection. In order to explore the regulatory role and molecular mechanism of NDR1 in IL-17-induced viral pneumonia inflammatory response, this study innovatively prepared viral pneumonia cell model by RSV virus infection, and explored its mechanism of action on congenital immune response to viral pneumonia by knockout and overexpression of NDR1 gene. In this study, IL-17 was used to induce the inflammatory response of cells, and NDR1 overexpression and NDR1 silencing vectors were prepared to regulate the expression of NDR1 gene in cells. By detecting the expression of inflammatory factors and signal molecules in cells, the mechanism of NDR1 gene in IL-17-induced innate immune response to viral pneumonia was explored, expected to provide a theoretical basis for finding new therapeutic targets for viral pneumonia.

## 2. Materials and methods

### 2.1 The subjects

Mouse alveolar monocytes RAW264.7 were purchased from American Type Culture Collection; TB Green® Premix Ex Taq™ II (Tli RNaseH Plus) and PrimeScript™ II 1st Strand cDNA Synthesis Kit were purchased from Takara, Japan; Dulbecco’s Modified Eagle Media (DMEM) high glucose medium, 0.25% trypsin, fetal bovine serum (FBS), and opti-MEM were purchased from Gibco, USA; Trizol reagent, thiazole blue (MTT), dimethyl sulfoxide (DMSO), JetPEI kit, and pcDNA3.1 were all purchased From Sigma, USA; Regulation of Investigatory Powers Act (RIPA) cell lysate, Protein Quantification Kit (BCA Assay), polyvinylidene fluoride membrane (PVDF), and Efficient chemiluminescence kit (ECL) were purchased from Shanghai Biyuntian Biotechnology Co., Ltd.

### 2.2 Cell culture and virus amplification

DMEM complete medium containing 10% fetal bovine serum was used to culture Hep-2 and RAW264.7 cells in a cell incubator with 5% CO_2_ at 37°C. When the cell confluence reached 80 ~ 90%, the cell started to passage. The cell surface was washed with PBS, and the cells were digested with 0.25% trypsin. After all the cells were detached, the cells were centrifuged at 1000 rpm for 10 minutes. After the pelleted cells were washed with PBS once, fresh complete medium was added to prepare a cell suspension, and the subculture was carried out at a ratio of 1:4.

The Hep-2 cells in the logarithmic phase were used for RSV amplification. The cells were inoculated in a 75 cm^2^ culture flask at a concentration of 5 × 10^5^ cells/mL. Then, the original medium was discarded when a monolayer of cells was formed. 5 mL virus solution was added then. The cells were shaken every 15 minutes, and maintenance solution containing 2% FBS was added. After 80% of the cells were affected, they were placed at −80°C and 37°C repeatedly for freezing and thawing. Next, they were centrifuged at 3000 rpm for 10 min. At this time, the supernatant was the virus solution, and it was stored at −80°C.

### 2.3 Determination of median infective dose (TCID50)

Hep-2 cells were inoculated in a 96-well plate, and DMEM medium was used to dilute the virus solution containing RSV to eight concentrations. Then, the original cell culture medium was discarded, and 100 μL of the diluted virus solution was added to each well. Next, the cells were shaken once every 15 minutes. The normal cells were used as a negative control. The cell fluid and virus fluid were discarded, and 200 μL of maintenance fluid containing 2% FBS was added to each well. When the cells with the highest dilution degree were not infected, the TCID50 was calculated as follows. $DR=\frac{A-50\%}{A-B}$, lgTCID50=C+DR×D (DR is the distance ratio; *A* is the percentage of the infection rate higher than 50%; *B* is the percentage of the infection rate lower than 50%; C is the logarithm of the diluted virus solution with an infection rate higher than 50%; and D is the logarithm of the dilution multiple).

### 2.4 Overexpression/silencing of cell NDR1

The full length of the CoDing Sequence (CDS) region of the NDR1 gene was amplified by PCR. After recovery, purification, and double digestion with NotI and AsiSI, it was connected to the pcDNA3.1 vector and transformed into DH5α competent cells. Then, the plasmid was extracted for double enzyme digestion and sequencing identification. Next, it was transfected into RAW264.7 cells.

The siRNA sequence of NDR1 (5ʹ-AGACCAGCUGCGAUAUCUAUUTT-3ʹ) and the negative control siRNA sequence (5ʹ-UUCUCCGAACGUGUCACGU-3ʹ) were designed. RAW264.7 cells were inoculated in a 12-well plate and cultured overnight. Then, siRNA mimics were dissolved in opti-MEM medium, and the final concentration was adjusted to 10 nM. Then, the transfection complex was added dropwise to the target cells to be transfected as per the JetPEI kit instructions. The medium was replaced after overnight culture. Next, the culture was continued for 36 hours.

### 2.5 Grouping and processing

There were five groups in the study, namely, blank group (blank), RSV-infected cell model group (model), negative siRNA transfection group (NC), overexpression NDR1 vector transfection group (OE), and NDR1 siRNA interference vector transfection group (SE).

RAW264.7 cells were inoculated in a 6-well plate. When the cell confluence reached about 80%, 500 μL RSV virus solution of 100 TCID 50/mL was added to the cell wells except the blank group. The cells were shaken once every 15 minutes. After 1 hour, the original virus solution was discarded, and 2.5 mL of 2% FBS maintenance solution was added to the blank group and model group, respectively. The NC group was transfected with a negative control siRNA vector, the OE group was transfected with an overexpression NDR1 vector, and the SE group was transfected with NDR1 siRNA interference vector, and 2.5 mL of maintenance solution containing 2% FBS was added. 36 h after transfection, 50 ng/mL IL-17 was used to stimulate for 3 h.

### 2.6 Cytokine determination

Cells in each group were stimulated with 50 ng/mL IL-17 at 36 h after transfection, and the cell supernatant was collected. The secretion levels of interleukin 6 (IL-6), interferon β (IFN-β), chemokine (C-X-C motif) ligand 2 (CXCL2), and chemokine (C-C motif) ligand 2 (CCL20), and other factors were detected using an Enzyme-linked immunosorbent assay (ELISA) kit. ELISA reagents were added in sequence, and the detection wells were washed with phosphate buffered saline tween (PBST). Then, the secondary antibody was added, followed by incubation at room temperature. After the detection wells were washed with PBST, horseradish peroxidase-conjugated anti-secondary antibody was added, followed by incubation at room temperature. Again, the PBST was used to wash the detection hole, and then the substrate was added, followed by incubation for 30 min in the dark. Next, the stop solution was added, and the optical density (OD) value was determined using the microplate reader to calculate the protein content.

### 2.7 Extraction of total cell RNA and RT-qPCR detection

The total RNA in the cell was extracted according to the Trizol method, and after the integrity and purity were identified, the reverse transcription kit was used to perform the reverse transcription of cDNA. With cDNA used as a template, the expression levels of NDR1, IL-6, tumor necrosis factor α (TNF-α), IFN-β, and β-actin were quantitatively detected according to the instructions of the TB Green® Premix Ex Taq™ II (Tli RNase H Plus) kit, where β-actin was the internal reference gene, and the relative expression level of the target gene was detected according to the 2^−ΔΔCt^ method. Table 1 shows the quantitative primer information.

**Table 1.** RT-qPCR primer information

Name	Sequence (5ʹ3ʹ)
NDR1	F: AAGGGCCATGTGAAACTTTCC
	R: CAGGAGTGCCCACTGTAGAGA
IL-6	F: GGCGGATCGGATGTTGTGAT
	R: GGACCCCAGACAATCGGTTG
TNF-α	F: GGGTGTTCATCCATTCTCTACC
	R: GTCCCAGCATCTTGTGTTTC
IFN-β	F: ACACCAGCCTGGCTTCCATC
	R: TTGGAGCTGGAGCTGCTTATCGTTG
RSV-F	F: TTGGATCTGCAATCGCCA
	R: CTTTTGATCTTGTTCACTTCTCCTTCT
TLR3	F: TGGATTCTTCTGGTGTCTTCC
	R: AGTTCTTCACTTCGCAACGC
β-actin	F: AGTGTGACGTTGACATCCGT
	R: GCAGATACGTAACAGTCCGC

### 2.8 Western blot

After the cells were treated for 48 hours, the cells were washed twice with phosphate buffer, and cell lysate was added to fully lyse the cells, followed by centrifugation at 12,000 rpm for 10 min at 4°C. Then, the supernatant was collected. The BCA protein quantification kit was used to detect and adjust the protein concentration. 5× sodium dodecyl sulfate-polyacrylamide gel electrohoresis (SDS-PAGE) was prepared and 50 μg protein was used for electrophoresis separation. Tris-Buffered Saline and Tween (TBST) solution containing 5% skimmed milk powder was used to block at room temperature for 1 h, and diluted NDR1 (1:500), extracellular signal-regulated kinase (ERK1/2, 1:500), p38 (1:500), human recombinant 1 (TBK1, 1:500), nuclear factor kappa-B (NF-κB, 1:600), and β-actin (1:500) primary antibody were added, followed by incubation overnight at 4°C. Then, the horseradish peroxidase-labeled secondary antibody was added, followed by incubation at room temperature for 1 hour. Finally, the ECL chemiluminescence kit was used for development, and the gray value of the target protein was then detected.

### 2.9 Statistical processing

The SPSS19.0 software was used to process the experimental data. The difference between the groups was compared by one-way analysis of variance, and the *Mean±s.d*. was used. *P < *0.05 indicated the statistically significant differences.

## 3. Results

In this study, RAW264.7 cells were infected with RSV virus to prepare a viral pneumonia cell model, and IL-17 was used to induce the cell immune response, and NDR1 gene was overexpressed and knocked out, respectively, to explore the mechanism of its action on the innate immune response of the cell model.

### 3.1 Proliferation characteristics of RSV-F in RAW264.7 cells

**Figure 1.**
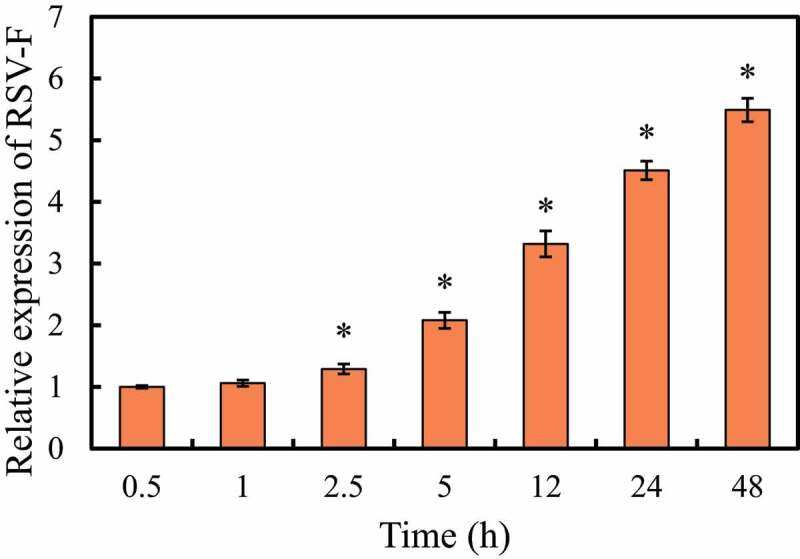
RSV-F mRNA expression in RAW264.7 cells at different times after RSV infection

Note: Compared with 0.5 h, *P < 0.05.

The expression of RSV-F mRNA in RAW264.7 cells after RSV infection was detected, and the results are shown in Figure 1. It was noted that, with the extension of the infection time, the expression of RSV-F mRNA in RAW264.7 cells showed a gradually increasing trend. Compared with infection for 0.5 h, the expression level of RSV-F mRNA in cells increased significantly after infection for 2.5 h to 48 h (*P < *0.05).

### 3.2 The effect of RSV infection on NDR1 expression

**Figure 2.**
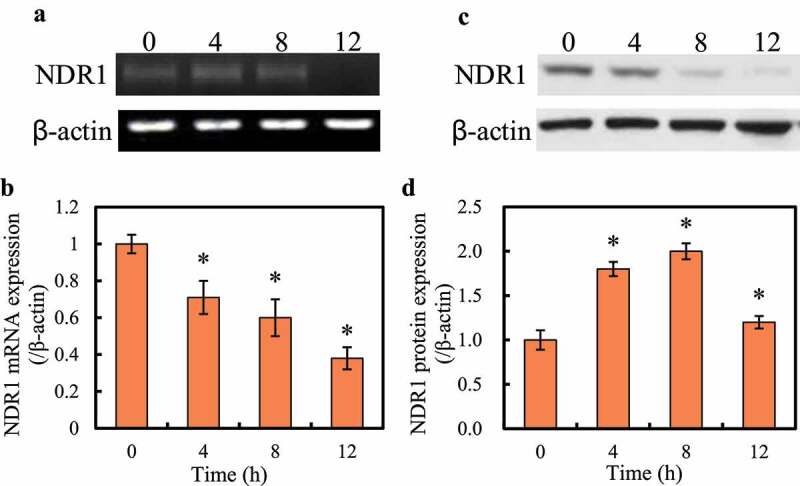
Changes of NDR1 expression in RAW264.7 cells at different times after RSV virus infection

Note: A is the map of mRNA agarose gel electrophoresis; B is the map of relative mRNA expression; C is the map of Western blot; and D is the map of relative protein expression. Compared with 0 h, *P < 0.05.

RT-qPCR and Western blot were performed to detect the changes in the expression of NDR1 gene in RAW264.7 cells after RSV virus infection, and the results are shown in Figure 2. It was noted that as the infection time increased, the expression levels of NDR1 mRNA and protein in RAW264.7 cells showed a gradual decline trend. Compared with 0 h, NDR1 mRNA and protein expression in RAW264.7 cells were significantly reduced after 4 h, 8 h, and 12 h of infection (*P < *0.05).

### 3.3 The secretion of cytokines and chemokines in RSV-infected cells

**Figure 3.**
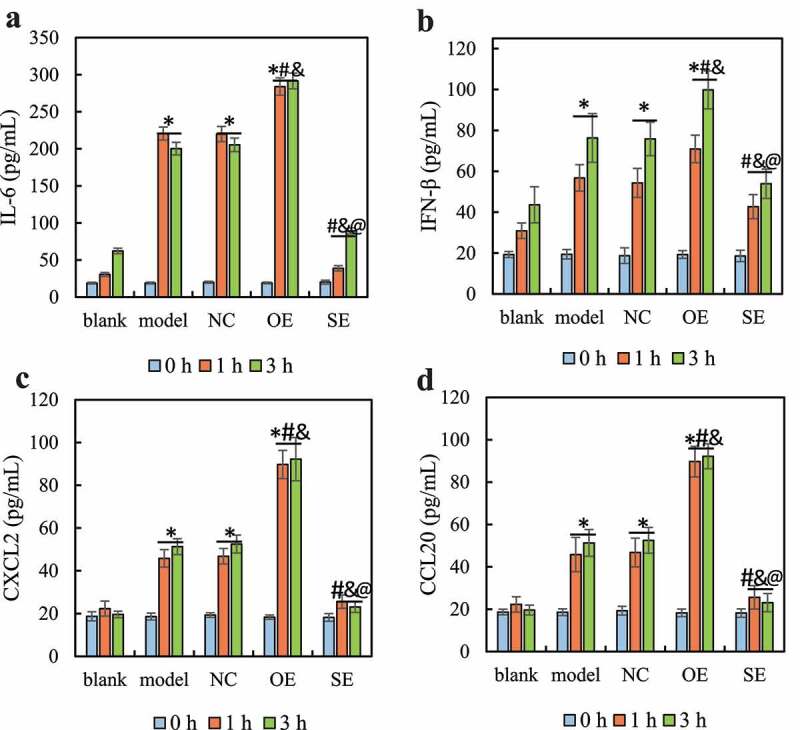
Changes in the secretion levels of cytokines and chemokines in RAW264.7 in each group

Note: A is the content of IL-6; B is the content of IFN-β; C is the content of CXCL2; D is the content of CCL20. Compared with blank group, *P < 0.05; compared with the model group, ^#^*P < *0.05; compared with the NC group, ^&^*P < *0.05; compared with the OE group, ^@^*P* < 0.05.

ELISA detected the differences in the secretion of cytokines and chemokines IL-6, IFN-β, CXCL2, and CCL20 in RAW264.7 cells after IL-17 stimulated for 0 h, 1 h, and 3 h. The results are shown in Figure 3. It was noted that, with the increase of stimulation time, the secretion levels of IL-6, IFN-β, CXCL2, and CCL20 factors in each group of cells showed an increasing trend. After IL-17 stimulation for 1 h and 3 h, compared with the blank group, the IL-6, IFN-β, CXCL2, and CCL20 secretion levels in the model group, NC group, and OE group were significantly increased (*P < *0.05). Compared with the model group and the NC group, the secretion levels of IL-6, IFN-β, CXCL2, and CCL20 in the cells of the OE group were significantly increased (*P < *0.05), while the *IL-6*, IFN-β, CXCL2, and CCL20 levels in the cells of the SE group were significantly down-regulated (*P < *0.05). Compared with the OE group, the secretion levels of IL-6, IFN-β, CXCL2, and CCL20 in the SE group were significantly down-regulated (*P < *0.05). There was no significant difference in the secretion levels of each factor between the model group and the *NC* group (*P > *0.05), and there was no significant difference between the blank group and the SE group (*P > *0.05).

### 3.4 Cellular inflammatory factors induced by IL-17

**Figure 4.**
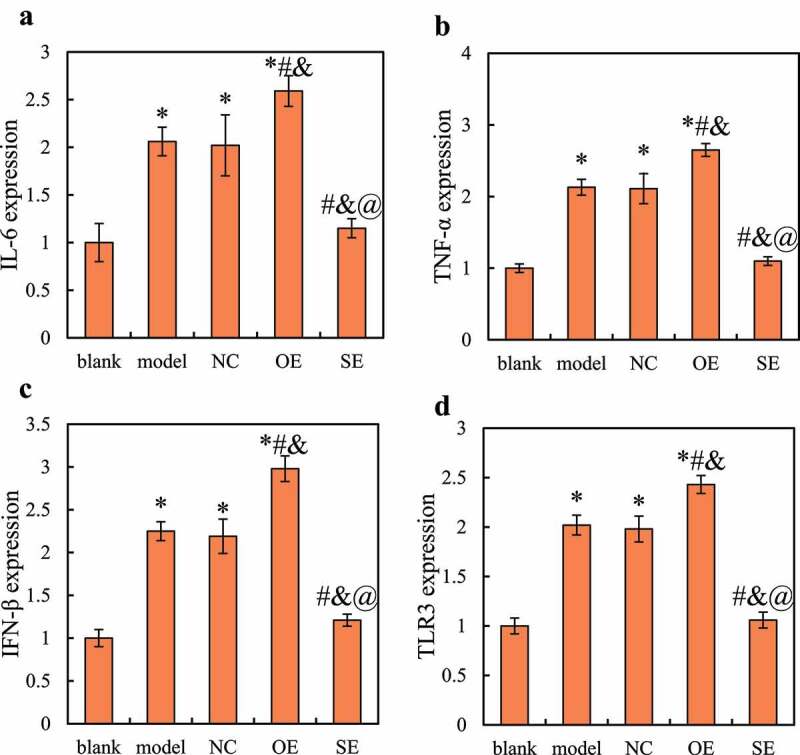
Changes in expression of inflammatory factors in RAW264.7 cells in each group

Note: A is the relative expression of IL-6; B is the relative expression of TNF-α; C is the relative expression of IFN-β; D is the relative expression of TLR3. Compared with blank group, *P < 0.05; compared with the model group, ^#^*P < *0.05; compared with the NC group, ^&^*P < *0.05; compared with the OE group, ^@^*P* < 0.05.

The NDR1 overexpression vector and siRNA interference vector were transfected into RAW264.7 cells, and the cells were infected with RSV for 3 hours. RT-qPCR was used to detect the expression of inflammatory cytokines in the cells. The results were shown in Figure 4. Compared with the blank group, the relative expression levels of IL-6, TNF-α, IFN-β, and TLR3 mRNA in the model group, NC group, and OE group were significantly up-regulated *(P < 0.05*); compared with the model group and NC group, the relative expression levels of IL-6, TNF-α, IFN-β, and TLR3 mRNA in OE group were significantly up-regulated (*P < *0.05), while the relative expression of IL-6, TNF-α, IFN-β, and TLR3 mRNA in SE group cells was significantly down-regulated (*P < *0.05); compared with the OE group, the relative expression levels of IL-6, TNF-α, IFN-β, and TLR3 mRNA in the SE group were significantly down-regulated (*P < *0.05). There was no significant difference in the expression levels of inflammatory factors between the model group and the NC group (*P > *0.05), and the expression levels of the inflammatory factors were not significantly different between the blank group and the SE group (*P > *0.05).

### 3.5 The activation of signal molecules induced by IL-17

**Figure 5.**
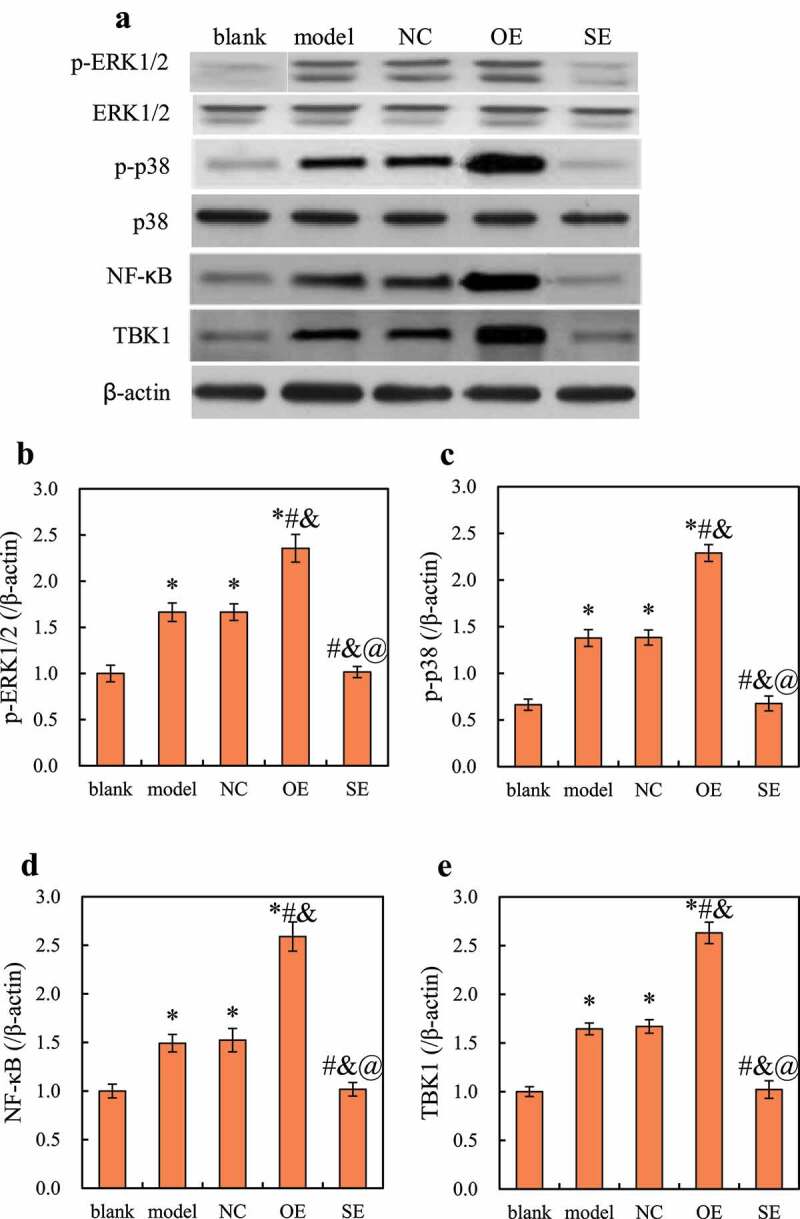
Changes in target gene protein expression in RAW264.7 cells in each group

Note: (a) is the western blot figure; (b) is the relative expression of p-ERK1/2; (c) is the relative expression of p-p38; (d) is the relative expression of NF-κB; (e) is the relative expression of TBK1. Compared with blank group, *P < 0.05; compared with the model group, ^#^*P < *0.05; compared with the NC group, ^&^*P < *0.05; compared with the OE group, ^@^*P* < 0.05.

Western blot detected the differences in the expression levels of target genes ERK1/2, p38, NF-κB, and TBK1 protein in each group of cells, and the results are shown in Figure 5(a). It was noted that, the protein expression levels of p-ERK1/2, p-p38, NF-κB, and TBK1 in the model group, NC group, and OE group were significantly increased, while the expression in SE group did not change much.

Then, the relative expression difference of the target gene was calculated, and the results are shown in Figure 5(b–e). Compared with the blank group, the relative expression levels of p-ERK1/2, p-p38, NF-κB, and TBK1 in the model group, the NC group, and the OE group were significantly up-regulated (*P < *0.05); compared with the model group and the NC group, the relative expression levels of p-ERK1/2, p-p38, NF-κB, and TBK1 in the cells of the OE group were significantly up-regulated *(P* < 0.05), while the expression of p-ERK1/2, p-p38, NF-κB, and TBK1 in the cells of the SE group was significantly down-regulated (*P < *0.05); *compared* with the OE group, the relative expression levels of p-ERK1/2, p-p38, NF-κB, and TBK1 in the SE group were significantly down-regulated (*P < *0.05). The target gene protein expression level of model group and NC group had no significant difference (*P > *0.05), and the target gene protein expression level of blank group and SE group had no significant difference (*P > *0.05).

## 4. Discussion

IL-17 can bind to the IL-17 receptor, which in turn induces the expression of cytokines and chemokines [11]. In this study, RSV was used to infect mouse alveolar monocytes RAW264.7, and IL-17 induced cells to obtain a viral pneumonia cell model. The results showed that, compared with normal RAW264.7 cells, the levels of IL-6, TNF-α, IFN-β, CXCL2, and CCL20 in the cells after RSV infection increased significantly. IL-6 is a multi-effect cytokine with a wide range of functions, which can participate in the regulation of cell growth and differentiation, and has functions, such as immune response and acute-phase response [12]. TNF-α is the initiator of inflammatory response. When TNF-α is released excessively, the balance of inflammatory factors and anti-inflammatory factors is realized [13]. Increased expression of IFN-β can help the body to clear the virus quickly, but excessive secretion can cause severe inflammation and cause damage to the body [14]. When pathogens invade the body, certain substances can cause the targeted chemotaxis of immune cells [15]. CXCL2 and CCL20 belong to the subfamily of chemokines. CXCL2 can induce the directional migration of inflammatory leukocytes to the damaged area, and CCL20 is a pro-inflammatory chemokine [16,17]. When the virus invades the body, the levels of CXCL2 and CCL20 increase, which will increase the inflammatory response at the site of inflammation [18]. The results of this study confirmed that RSV can induce inflammation in RAW264.7 cells after infecting RAW264.7 cells, thus successfully preparing a viral pneumonia cell model.

NDR1 participates in the regulation of a variety of cell biological processes and can participate in the regulation of cell signal transduction, such as TGF-β, STAT1, and other signal pathways [19,20]. NDR family genes are involved in biological processes such as viral infection. Studies have shown that when pathogenic microorganisms invade the body, the induced increase of NDR1 expression level can effectively resist the infection of pathogenic microorganisms [21]. Liu et al. (2018) studied the viral infection model prepared by interferon stimulation and induction, and the results showed that mice with deletion of NDR1 gene were more susceptible to viral and bacterial infection [20]. Liu et al. (2019) confirmed that NDR2 gene can participate in the innate immunity mechanism of antiviral in the body [22]. In order to further verify the regulatory effect of NDR1 on the immune response of IL-17-induced viral pneumonia cell models, NDR1 overexpression and interference vectors were prepared and transfected into RSV-infected and IL-17-induced RAW264.7 cells. The results showed that overexpression of NDR1 can enhance the secretion or expression of the above-mentioned inflammatory factors and chemokines, while silencing NDR1 can inhibit the secretion or expression of inflammatory factors and chemokines caused by RSV infection and IL-17 induction. It showed that NDR1 can positively regulate the antiviral natural immune response.

Il-17 is an important cytokine that induces tissue inflammation and is involved in a variety of autoimmune diseases. However, knockout of NDR1 gene can inhibit IL-17-induced cytokine expression, indicating that NDR1 is involved in regulating the process of autoimmune diseases [23]. Subsequently, the molecular mechanism of NDR1 regulating IL-17-induced viral pneumonia antiviral natural immune response was further studied. Western blot technology was used to detect changes in the protein expression of ERK1/2, p38, NF-κB, and TBK1. The ERK1/2 signaling pathway is mainly involved in the regulation of cell proliferation and differentiation and is closely related to the transcription and expression of certain genes. This signaling pathway is also related to the inflammatory response [24]. The p38 MAPK pathway can activate the secretion of pro-inflammatory factors, such as TNF-α and IL-1 [25]. NF-κB can participate in the response of cells to external stimuli, and plays a key role in cellular inflammatory response and immune response [26]. The results of the study showed that interference with the expression of NDR1 in RSV-infected RAW264.7 cells can inhibit IL-17-induced activation of ERK1/2, p38, NF-κB, and TBK1 signaling molecules, and attenuate the expression of cytokines, inflammatory factors, and chemotaxis. It showed that NDR1 positively regulates IL-17 signaling pathway and can treat IL-17 signaling pathway-related viral pneumonia by regulating ERK1/2, p38, NF-κB, and TBK1 signaling molecules.

## 5. Conclusion

This study only verifies the molecular mechanism of NDR1 regulating IL-17-induced viral pneumonia at the in vitro cellular level. In the follow-up, in vivo animal experiments are needed to further explore the role of NDR1 in regulating IL-17-induced infectious diseases or autoimmune diseases, so as to provide reference for using NDR1 as a new target for the treatment of this disease.
